# Pathological and genomic phenotype of second neuroendocrine carcinoma during long-term follow-up after radical radiotherapy for nasopharyngeal carcinoma

**DOI:** 10.1186/s13014-021-01898-z

**Published:** 2021-10-11

**Authors:** Ying-peng Peng, Qiao-dan Liu, Yu-jing Lin, Shun-li Peng, Rong Wang, Xi-wei Xu, Wei Wei, Gui-hua Zhong, Yu-ling Zhou, Ya-qin Zhang, Ye Liu, Si-yang Wang, Hai-yu Hong, Zhi-gang Liu

**Affiliations:** 1 The Cancer Center of the Fifth Affiliated Hospital of Sun Yat-sen University, Zhuhai, 519000 Guangdong Province China; 2 Guangdong Provincial Key Laboratory of Biomedical Imaging, The Fifth Affiliated Hospital, Sun Yat-sen University, Zhuhai, 519000 Guangdong Province China; 3 Department of Pathology, The Fifth Affiliated Hospital of Sun Yat-Sen University, Zhuhai, 519000 Guangdong Province China; 4 Department of Radiology, The Fifth Affiliated Hospital of Sun Yat-sen University, No. 52 Meihua Dong Road, Zhuhai, 519000 Guangdong Province China; 5 Allergy Center, Department of Otolaryngology, The Fifth Affiliated Hospital of Sun Yat-sen University, No. 52 Meihua Dong Road, Zhuhai, 519000 Guangdong Province China

**Keywords:** Radiation-induced neoplasms, Neuroendocrine carcinoma, Nasopharyngeal carcinoma, Radiotherapy, Mutational analysis

## Abstract

**Background:**

Second head and neck neuroendocrine carcinoma (NEC) after radical radiotherapy for nasopharyngeal carcinoma (NPC) treatment is rarely reported. The prognosis of second cancer is poor, and our research focuses on finding a breakthrough in the treatment. In this study, we aimed to investigate clinicopathological characteristics and to identify the genomic landscape of second head and neck NECs.

**Methods:**

We collected five second head and neck NEC cases in the recent three years from our patient database. Clinicopathological data and images were obtained. Genomic analysis was performed using high-throughput second generation sequencing. KEGG pathway enrichment analyses between high-frequency mutations were performed using the STRING database.

**Results:**

All patients had been diagnosed with second NEC, according to the pathological observations. The interval between diagnosis of NPC and NEC ranged from 10 to 18 years. Two patients had brain or liver metastasis at three and nine months, respectively, after the diagnosis of NEC. Three patients died of the disease with the overall survival time ranging from three to nine months. Commonly altered genes (50%) in second head and neck NECs included *TP53, RB1, NOTCH2, PTEN, POLG, KMT2C, U2AF1, EPPK1, ELAC2, DAXX, COL22A1*, and *ABL1.* Those genetic lesions might affect p53 signaling, MAPK signaling, PI3K-Akt signaling, sphingolipid signaling, and neurotrophin signaling pathways.

**Conclusions:**

Second head and neck NECs had poor prognosis. We revealed, for the first time, the mutational landscape, high-frequency somatic mutations, and potential signaling pathways of second head and neck NECs. Its optimal treatment model needs to be further studied in future clinical trials.

## Background

Nasopharyngeal carcinoma (NPC) mainly occurs in east and southeast Asia [1]. The most common site for NPC is the pharyngeal recess, and squamous cell carcinoma is its most common type, accounting for more than 95% of NPC cases [2]. The primary treatment for NPC is radiotherapy, and for locally advanced NPC, radiotherapy combined with chemotherapy is the standard treatment [3]. For radical radiotherapy, the clinical study RTOG0225 recommended the Intensity Modulated Radiation Therapy (IMRT) [4]. Long-term results after IMRT showed that the 5-year local recurrence rate of NPC reduced to 7.4%, and the 5-year overall survival rate increased to 82.0%  [5]. Concomitantly, the development of radiation-induced carcinomas has become one of the serious complications; the most common types are squamous cell carcinomas and sarcomas.

Second neuroendocrine carcinoma (NEC) of head and neck is rare, especially of the nasopharynx [6–8]. To our knowledge, there have been only a dozen NEC cases reported with a history of radiotherapy for NPC [9–12]. Very little research has been done on genetic alterations of radiation induced NECs. In particular, the mutational landscape of second head and neck NECs has not been studied before. NEC can be classified as large cell NEC, small cell NEC, typical carcinoid and atypical carcinoid. Poorly differentiated NECs of the nasopharynx are very aggressive and have a high recurrence and metastasis rate [13–16]. There is currently no effective treatment, thus research into the mechanisms of NEC and possible therapy targets is vital.

In this study, we identified the clinicopathologic features of five NEC patients with NEC in the high incidence area of NPC, who had received radical radiotherapy for NPC before. We also investigated their mutational characteristics, hoping to find potential targets for the treatment of second head and neck NECs.

## Methods

### Sample and data collection

We collected all patients with NPC (N = 1052) in our hospital from June, 2017 to September, 2020. Then we selected five patients who met the criteria of radiation-induced head and neck NEC (second NEC). All patients had a history of radical radiotherapy for NPC. Clinical data, such as clinicopathologic features, treatments, and outcomes were extracted from medical records. The TNM status of each tumor was reclassified according to the criteria of the American Joint Committee on Cancer (2008). Radiographic and nasal endoscopy images were exported from our medical image system. Magnetic resonance imaging (MRI) and nasal endoscopy images at diagnosis and during the follow-up of NEC were compared.

The paraffin blocks of the original NPC biopsy specimens, and the second NEC surgical specimens were sectioned for histopathological and immunohistochemical staining. Samples were taken and evaluated by the Department of Pathology in our hospital. Among these five patients, three of them had no valid data regarding NPC specimens due to storage timeout or exhaustion of paraffin blocks.

The paraffin blocks of the second NEC specimens were collected for genetic testing. Among these five patients, one did not undergo genetic testing for personal reasons.

Based on previous studies [10], the criteria for the diagnosis of radiation induced neoplasms include (1) a history of radiation therapy is required and the tumor is induced in the radiation target area, (2) it takes a long latent period (at least two years) from the end of radiotherapy to the onset of the induced tumor, (3) there must be a different histopathological pattern between the induced tumor and the primary tumor to rule out metastasis or recurrence.

### Hematoxylin and eosin (H&E) staining and immunohistochemistry

Paraffin sections were stained for CD56, EBER, SYN, CGA, P40, and CK5/6. Images were acquired after H&E and immunohistochemical staining. The immunohistochemical staining were scored by experienced pathologists based on the intensity and percentage [17]. The staining intensity was scored as 1 (light-yellow), 2 (brown-yellow), 3 (brown). The staining percentage was scored as 1 (0–25%), 2 (26–50%), 3 (51–75%), 4 (76–100%). The final score was defined as the intensity score multiplied by the percentage score. Finally, the expression was identified as negative (−) if the final score was 0, weakly positive (+) if the final score was between 1 and 4, moderately positive (++) if the final score was between 5 and 8, and strongly positive (+++) if the final score was between 9 and 12.

### High-throughput second generation sequencing and genetic alteration detection

Based on current scientific research and clinical data, samples were tested for 688 genes associated with the development, diagnosis, treatment, and prognosis of solid tumors at the Beijing Genomics Institute (BGI) using high-throughput second generation sequencing.

### Mutational analysis and pathway enrichment analysis

Second NEC somatic landscape, also named Oncoplot, which contains somatic driven-gene mutation types and frequency, was visualized using R Bioconductor packages, “GenVisR” and “reshape2”. Protein interaction and KEGG pathway enrichment analyses of high-frequency mutations were performed using the STRING database (https://string-db.org/).

### Statistical analysis

Survival times were obtained through imaging and telephone follow-up. The end time of follow-up was defined as 2020/09/26. Progression free survival (PFS), from the beginning of the treatment to progression or death, and overall survival (OS), from the beginning of treatment to death, were recorded and used to assess patients’ survival time.

## Results

### Poor prognosis of radiation-induced head and neck NEC

**Fig. 1:**
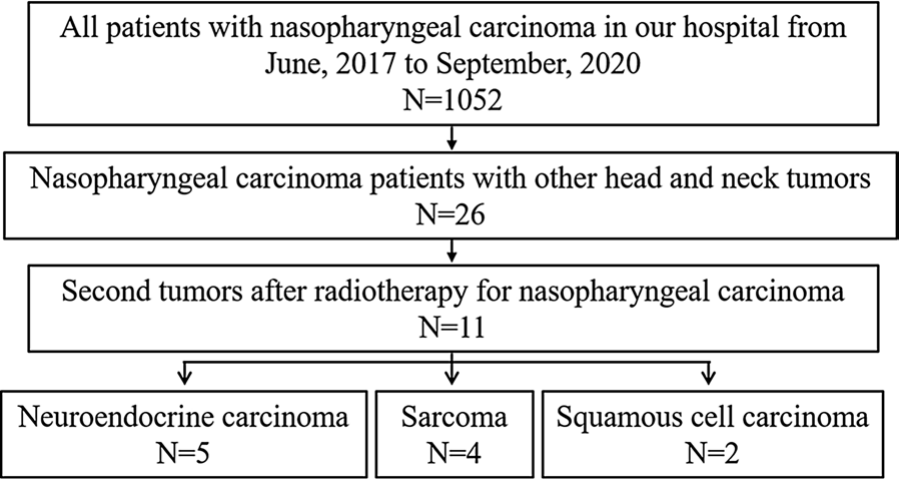
Flow chart of patient selection

We collected all patients with NPC (N = 1052) in our hospital from June, 2017 to September, 2020. Then we found 26 NPC patients with other head and neck tumors.11 of them were patients with second tumors after radiotherapy for NPC, as shown in Fig. 1. Of the 11 patients, five were diagnosed with NEC, four with sarcoma, and two with squamous cell carcinoma (NPC recurrence excluded by immunohistochemistry). The clinical characteristics of five NEC patients are summarized in Table 1. All patients were 49–67-year-old males. Patients had been diagnosed with NPC, stages from T2N0M0 to T3N2M0, with poorly differentiated or undifferentiated squamous carcinoma. All patients received radical radiotherapy and regular follow-up. The interval between diagnosis of NPC and NEC ranged from 10 to 18 years. The sites of second NEC were left nasal cavity, right hard palate, left nasal cavity, right nasal cavity-skull base, and left ethmoidal sinus, which were all within the radiation area. The patients were diagnosed with small cell NEC and received surgery and/or radiotherapy combined with chemotherapy. Three of five patients received re-irradiation for NEC. Patient 1 and Patient 3 had poor prognosis because of disease progression. The response was PD six months and two months respectively after RT. The response of Patient 4 was SD three months after RT. The main toxicities of radiotherapy were skin and mucosal related reactions, sore throat, bone marrow suppression, irritating cough, loss of appetite, etc. Two of them had brain or liver metastasis at three and nine months, respectively, after diagnosis of NEC. Three of them died of the disease, with the OS ranging from three to nine months. The other two patients were still alive at the time of writing this study, with a follow-up period of five months and one month.

**Table 1:** Clinical characteristics of five neuroendocrine carcinoma patients with a history of radical radiotherapy for nasopharyngeal carcinoma

		Patient 1	Patient 2	Patient 3	Patient 4	Patient 5
Age		59	67	50	56	49
Gender		Male	Male	Male	Male	Male
Year diagnosed NPC		2008	2007	2005	2010	2002
NPC stage		T3N2M0	T3N1M0	T3N0M0	T2N0M0	*
*Treatment of NPC*						
RT		2008	2007	2005	2010	2002
		70 Gy/35F	70 Gy/35F	Radical RT	70 Gy/35F	Yes*
		2D	2D	2D	2D	2D
CT		DDP	DDP	DDP	DDP	Yes*
Date diagnosed NEC		2018/09/20	2020/03/09	2019/01/31	2020/04/29	2020/09/01
Sites of NEC		Left nasal cavity	Right hard palate	Left nasal cavity	Right nasal cavity-skull base	Left ethmoidal sinus
*Treatment of NEC*						
ST		–	2020/03/06	2019/02/19	2020/04/23	2020/08/24
RT		GTV 70 Gy/33F	–	GTV 66 Gy/30F	GTV 64 Gy/30F	–
		2018/10/23	–	2019/04/08	2020/05/25	–
		IMRT	–	IMRT	IMRT	–
CT		VP	VP	VP	VP	GP
PFS		6 m	3 m	4 m	Censored	Censored
OS		9 m	3 m	9 m	Alive	Alive

NEC, neuroendocrine carcinoma; NPC, nasopharyngeal carcinoma; OS, overall survival; IMRT, intensity modulated radiation therapy; RT, radiotherapy; CT, chemotherapy; ST, surgery therapy; GP, gemcitabine + DDP (cisplatin); VP, VP16 (etoposide) + DDP (cisplatin); 2D, two-dimensional radiotherapy

–, not performed; *, no exact details

### Histopathological and immunohistochemical features of radiation-induced head and neck NECs

**Fig. 2:**
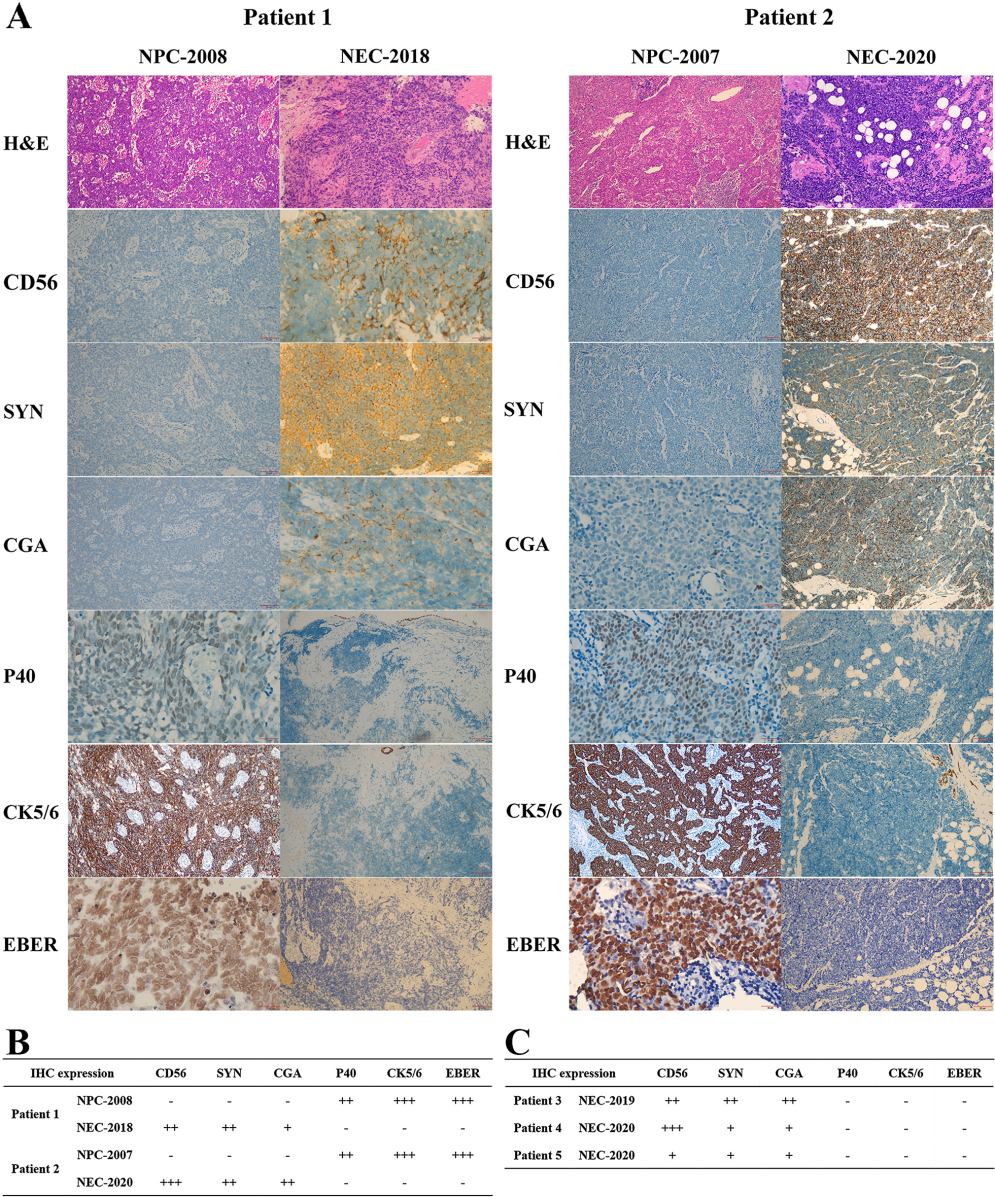
Histopathological and immunohistochemical features of five cases. **A** Histopathological and immunohistochemical images of NPC vs. NEC for Patient 1 and Patient 2. **B** NPC and NEC IHC expression for Patient 1 and Patient 2. **C** NEC IHC expression for Patient 3, Patient 4 and Patient 5. *NPC* nasopharyngeal carcinoma, *NEC* neuroendocrine carcinoma, *IHC* immunohistochemistry

We compared the H&E and immunohistochemical expression of the original NPC specimens to the second NEC specimens of Patient 1 and Patient 2; as shown in Fig. 2A, B. Patient 1 was pathologically diagnosed with nasopharyngeal undifferentiated non-keratinizing carcinoma in 2008 (NPC-2008) and left nasal cavity high-grade NEC in 2018 (NEC-2018). Patient 2 was pathologically diagnosed with nasopharyngeal poor differentiated squamous carcinoma in 2007 (NPC-2007) and right hard palate small cell NEC in 2020 (NEC-2020). Immunohistochemistry (IHC) expression for CD56, EBER, SYN, CGA, P40, and CK5/6 were performed by semi-quantitative analysis. The NPCs were strongly positive (+++) for EBER and CK5/6, and moderately positive (++) for P40, whereas the NECs were moderately positive (++) or strongly positive (+++) for CD56 and SYN, and weakly positive (+) or moderately positive (++) for CGA. Also, we analyzed NEC IHC expression of Patient 3, Patient 4 and Patient 5 (Fig. 2C). It showed that CD56, SYN, CGA expression were all positive on different levels, whereas EBER, CK5/6, P40 were all negative; therefore, it supported the diagnosis of NEC.

### Local progression or distant metastasis in radiation-induced head and neck NECs

**Fig. 3:**
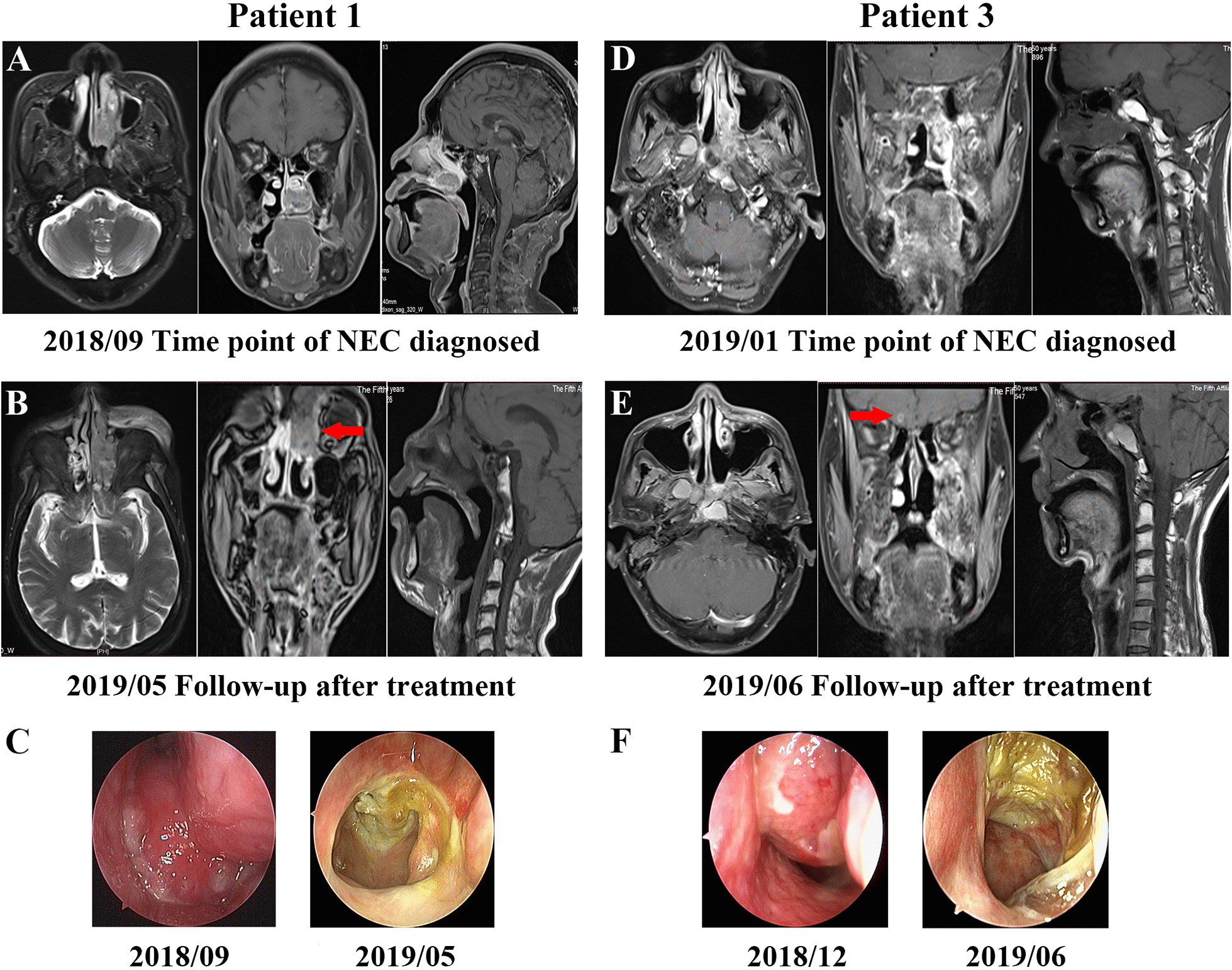
MRI and nasal endoscopy images at diagnosis and during the follow-up of NEC of Patient 1 and Patient 3. **A** MRI images of patient 1 at diagnosis. **B** MRI images of Patient 1 during the follow-up. Progression in the upper bound of the tumor during the follow-up of Patient 1 (red arrow). **C** Nasal endoscopy images at diagnosis (2018/09) and during the follow-up (2019/05) of Patient 1. **D** MRI images of patient 3 at diagnosis. **E** MRI images of Patient 3 during the follow-up. Brain metastasis during the follow-up of Patient 3 (red arrow). **F** Nasal endoscopy images at diagnosis (2018/12) and during the follow-up (2019/06) of Patient 3

MRI and nasal endoscopy images at diagnosis and during the follow-up of NEC are shown in Fig. 3. For Patient 1, the tumor was in the left nasal cavity, mainly in the lower nasal cavity. The lesion in the lower nasal cavity disappeared in nasal endoscopy five months after chemoradiotherapy; however, there was an increase in the upper bound of the tumor according to the MRI (2019/05, red arrow). For Patient 3, the tumor was mainly in the left middle nasal cavity and it was connected with the thickened left lateral wall and the posterior wall of nasopharyngeal mucosa. The size of the tumor was approximately 33 mm × 17 mm × 24 mm. The patient underwent the surgery and chemoradiotherapy for NEC. One month after the treatment, the lesion in the nasopharynx and left nasal cavity was not seen in the MRI or nasal endoscopy. However, brain metastasis was observed through MRI (2019/06, red arrow).

### Commonly mutated genes and pathways of radiation-induced head and neck NECs

**Fig. 4:**
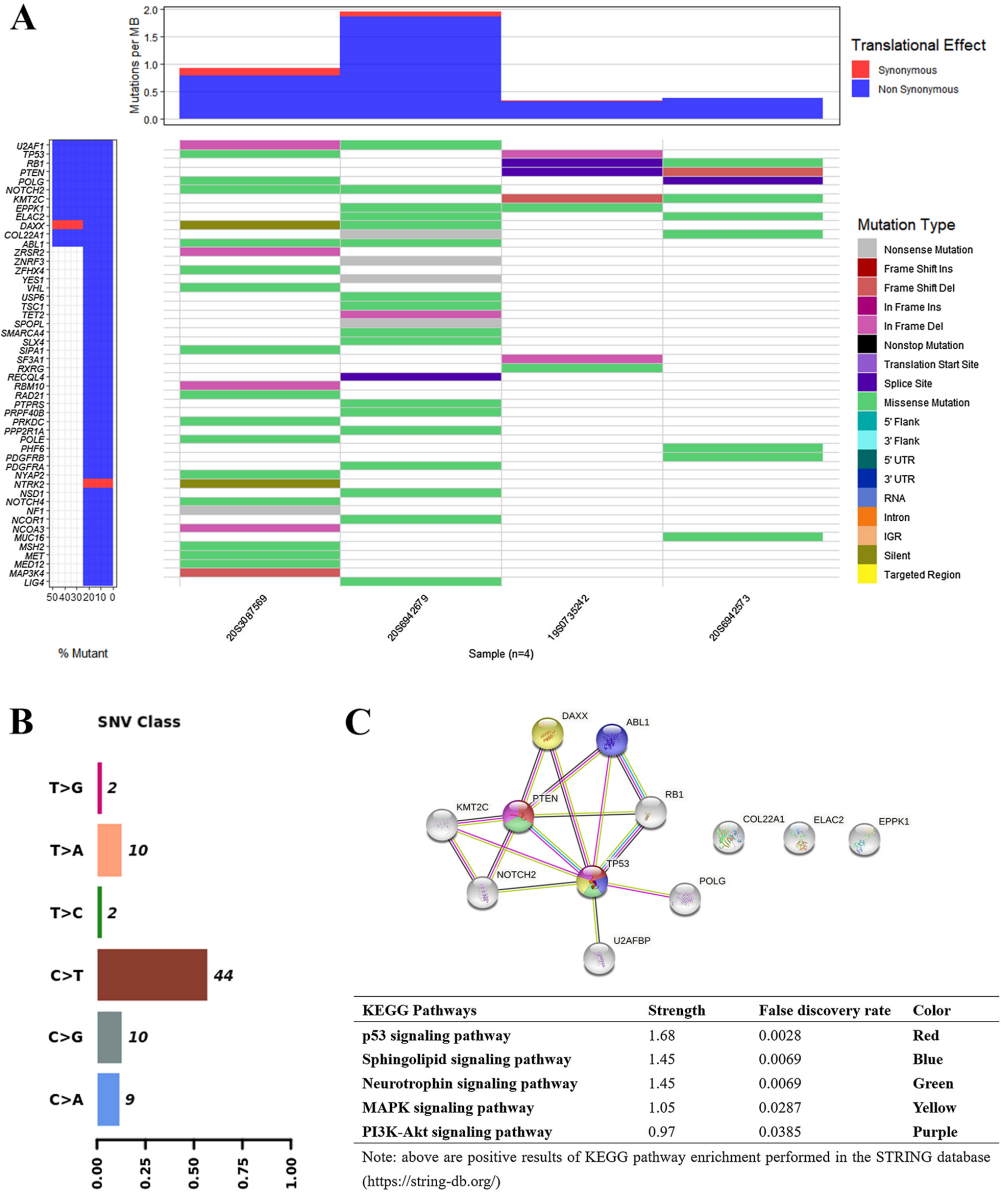
Genomic landscape of four second head and neck NEC cases. **A** Oncoplot of mutations. The tumor mutation burden, TMB, is shown on the top panel. The TOP 50 mutated genes and proportions are shown in left panels. Specific mutation types are displayed in different colors and shown in the right panel. *Notes*: Patient 2_20S3087569, Patient 3_19S0735242, Patient 4_20S6942679, Patient 5_20S6942573. **B** SNV class of mutations. **C** Protein interaction and KEGG pathway enrichment analyses between high-frequency mutations

We analyzed the data of four cases and showed the TOP 50 mutated genes and their frequency (Fig. 4A). Two out of four cases had *TP53, RB1, NOTCH2, PTEN, POLG, KMT2C, U2AF1, EPPK1, ELAC2, DAXX, COL22A1*, or *ABL1* mutations. The single-nucleotide variant (SNV) class was dominated by C>T (Fig. 4B). KEGG pathway enrichment analysis showed radiation-induced head and neck NECs might affect p53 signaling, MAPK signaling, PI3K-Akt signaling, sphingolipid signaling, and neurotrophin signaling pathways (Fig. 4C). It reveals a mutational signature different from that of NPC, which includes genetic lesions mainly affecting chromatin modification, ERBB-PI3K signaling and autophagy machinery [18], or NF-κB pathway of recurrent NPC [19]. All four cases had tumor mutation load of < 2 mutations per megabase (Mb).

## Discussion

Patients with NPC have long-time survival after radical radiotherapy; Therefore, second carcinoma has become an interesting and important issue to study. Radiotherapy is an important cause of second cancer, also called radiation-induced neoplasms. Based on previous studies, squamous cell carcinoma and sarcoma are the main pathologic types of second cancers in the head and neck [20–23], whereas NEC is rare. However, we identified more NEC cases than sarcoma or squamous cell carcinoma cases. That is different from previous literature. In our study, the radiotherapy for NPC of these five patients was 2D radiotherapy, with a large irradiation range. The nasal cavity, hard palate, and ethmoidal sinus received a certain dose of radiation, not only the skull base or nasopharynx. Besides, it took a long latency period from the end of radiotherapy to the onset of second NEC; different histopathological patterns were existed between the induced NEC and the primary NPC. According to the diagnostic criteria of RIN put forward by previous studies, all patients included in our study met the diagnosis of RIN.

We reported the phenotype of five patients with head and neck NECs, who had a history of radical radiotherapy for the treatment of NPC. All five patients were male, which was in accordance with previous studies that indicated that head and neck NEC occurs predominantly in male patients. After concurrent radiotherapy with or without adjuvant chemotherapy, NPC lesions cured. However, the patients developed second NECs within the irradiation area 10–18 years later; with different pathological features than those of NPC. Re-irradiation treatment for most second NECs were not effective but caused side effects. Unfortunately, they soon had aggressive local invasion or distant metastasis and three of the patients died of NEC despite receiving comprehensive treatment. Though we only identified five cases, that was the result of statistical data of NPC radiotherapy induced tumor in our hospital in the recent 3 years. In addition, the occurrence of radiation induced head and neck NEC is rare. That is a very special, tough group worthy of attention that has no standard treatment. That is also the limitation and specificity of our study.

Poorly differentiated second NEC has an extremely poor prognosis. Chen reported 11 patients with second primary malignancies after curative radiotherapy for NPC and found that the OS of patients with NEC was only three and four months (2/11), whereas that of patients with squamous cell carcinoma was 11–63 months (9/11) [9]. Wang reviewed 18 patients with NEC arising from the sinonasal tract, of which eight were post-irradiated NEC and 10 were primary ones [10]. The 5-year OS rates of post-irradiated NEC and primary NEC were 62.5 and 70% (*P* = .08), respectively. Because of the rare occurrence of second head and neck NECs, there is a lack of large studies on the survival time of these patients. However, it is widely recognized that second head and neck NECs often have local progression or distant metastasis. Our study was in accordance with previous onesas it showed that patients have a poor OS time (three to nine months). Thus, further studies on the molecular biological behavior and genetic background of NECs are essential to lay a foundation for advanced treatment strategies.

It is well known that Epstein–Barr virus (EBV) is closely related to NPC, and other nasopharyngeal malignancies, such as NK/T-cell lymphoma [24, 25]. Large cell NEC of the nasopharynx was also reported to be associated with EBV [26]. EBV-positive large cell NEC was shown to be sensitive to chemoradiotherapy and might have better prognosis [27]. However, the relationship between EBV and second NEC prognosis is unclear. EBV-encoded small RNA (EBER) is widely accepted to as an indicator of EBV infection [28]. In our study, four patients were positive for primary NPCs and negative for induced NECs, according to the detected EBER status, which means the tumor microenvironment and biological behavior might be completely altered in induced NECs. There was no EBER expression in second NECs; therefore, it is worth further exploring whether the corresponding pathological transformation had occurred.

The occurrence of radiation induced NEC is rare and has been rarely reported in previous literature. There is currently no report on genomic profiles of head and neck NECs, including second head and neck NECs. We are the first to perform the genetic analysis of NPC radiotherapy induced head and neck NECs. *TP53* and *RB1* were confirmed as characteristic genetic mutations in both small cell lung cancer (SCLC) and pulmonary large cell NEC [29–31]. In our study, commonly altered genes (50%) in second head and neck NECs included *TP53, RB1, NOTCH2, PTEN, POLG, KMT2C, U2AF1, EPPK1, ELAC2, DAXX, COL22A1*, and *ABL1*. Though limited by the small sample size, *TP53* and *RB1* mutations were consistent with lung NECs. Other genetic mutations, such as *NOTCH* and *KMT2C* may be useful for the treatment. NOTCH signaling pathway has been confirmed to be correlated with the occurrence, development, and prognosis of SCLC [32, 33]. SCLC cells with activation of NOTCH signaling may benefit from NOTCH pathway inhibitors combined with chemotherapy [33]. Retrospective analysis showed that NOTCH signaling pathway may be a potential biomarker for the immunotherapy in non-small cell lung cancer (NSCLC) [34]. KMT2C and KMT2D usually form complexes responsible for the methylation of H3K4.

It was found that *KMT2C/2D* mutations were accompanied by high mutation rate of tumor suppressor gene *TP53* and oncogene *KRAS*. The specific deletion of KMT2C/2D may promote the initiation of TP53/KRAS-induced lung cancer [35]. *KMT2C/2D* mutations were also reported to be closely associated with MSI-H and higher TMB. Colorectal cancer patients with *KMT2C/2D* mutations may benefit from the immune checkpoint inhibitor treatment [36]. Therefore, whether head and neck NECs with *NOTCH* or *KMT2C* mutations would be beneficial for immunotherapy is worth exploring.

## Conclusions

Second head and neck NECs had poor prognosis. We revealed, for the first time, the mutational landscape, high-frequency somatic mutations, and potential signaling pathways of second head and neck NECs. We believe our study may pave the way for finding potential therapeutic targets for the treatment of second head and neck NECs.

## Data Availability

The datasets used and/or analyzed during the current study are available from the corresponding author on request.

## References

[1] Bray F, Ferlay J, Soerjomataram I, Siegel RL, Torre LA, Jemal A (2018). Global cancer statistics 2018: GLOBOCAN estimates of incidence and mortality worldwide for 36 cancers in 185 countries. Cancer J Clin.

[2] Wang HY, Chang YL, To KF, Hwang JS, Mai HQ, Feng YF, Chang ET, Wang CP, Kam MK, Cheah SL, Lee M, Gao L, Zhang HZ, He JH, Jiang H, Ma PQ, Zhu XD, Zeng L, Chen CY, Chen G, Huang MY, Fu S, Shao Q, Han AJ, Li HG, Shao CK, Huang PY, Qian CN, Lu TX, Li JT, Ye W, Ernberg I, Ng HK, Wee JT, Zeng YX, Adami HO, Chan AT, Shao JY (2016). A new prognostic histopathologic classification of nasopharyngeal carcinoma. Chin J Cancer.

[3] Chen YP, Chan ATC, Le QT, Blanchard P, Sun Y, Ma J (2019). Nasopharyngeal carcinoma. Lancet.

[4] Lee N, Harris J, Garden AS, Straube W, Glisson B, Xia P, Bosch W, Morrison WH, Quivey J, Thorstad W, Jones C, Ang KK (2009). Intensity-modulated radiation therapy with or without chemotherapy for nasopharyngeal carcinoma: radiation therapy oncology group phase II trial 0225. J Clin Oncol.

[5] Mao YP, Tang LL, Chen L, Sun Y, Qi ZY, Zhou GQ, Liu LZ, Li L, Lin AH, Ma J (2016). Prognostic factors and failure patterns in non-metastatic nasopharyngeal carcinoma after intensity-modulated radiotherapy. Chin J Cancer.

[6] Vandist V, Deridder F, Waelput W, Parizel PM, Van de Heyning P, Van Laer C (2010). A neuroendocrine tumour of the sphenoid sinus and nasopharynx: a case report. B-ENT.

[7] Deviprasad S, Rajeshwari A, Tahir M, Adarsha TV, Gangadhara S (2008). Small-cell neuroendocrine carcinoma originating from the lateral nasopharyngeal wall. Ear Nose Throat J.

[8] Weinreb I, Perez-Ordoñez B (2007). Non-small cell neuroendocrine carcinoma of the sinonasal tract and nasopharynx. Report of 2 cases and review of the literature. Head Neck Pathol.

[9] Chen CL, Hsu MM (2000). Second primary epithelial malignancy of nasopharynx and nasal cavity after successful curative radiation therapy of nasopharyngeal carcinoma. Hum Pathol.

[10] Wang CP, Hsieh CY, Chang YL, Lou PJ, Yang TL, Ting LL, Ko JY (2008). Postirradiated neuroendocrine carcinoma of the sinonasal tract. Laryngoscope.

[11] Lin CH, Chiang TP, Shum WY, Hsu CH, Tsai YC, Tsao TY, Su CC (2009). Primary small cell neuroendocrine carcinoma of the nasal cavity after successful curative therapy of nasopharyngeal carcinoma: a case report. Kaohsiung J Med Sci.

[12] Abrigo JM, King AD, Leung SF, Vlantis AC, Wong JK, Tong MC, Tse GM, Ahuja AT (2009). MRI of radiation-induced tumors of the head and neck in post-radiation nasopharyngeal carcinoma. Eur Radiol.

[13] Cai Z, Lin M, Blanco AI, Liu J, Zhu H (2019). Epstein–Barr virus-positive large cell neuroendocrine carcinoma of the nasopharynx: report of one case and review of the literature. Head Neck Pathol.

[14] Bhardwaj N, Kakkar A, Irugu DVK (2018). Small cell neuroendocrine carcinoma: a rare nasopharyngeal malignancy with aggressive clinical course. Indian J Otolaryngol Head Neck Surg.

[15] Elloumi F, Fourati N, Siala W, Ghorbell L, Jlidi R, Ghorbel A, Frikha M, Daoud J (2014). Large cell neuroendocrine carcinoma of the nasopharynx: a case report. Cancer Radiother.

[16] Guo C, Pan Q, Su M, Li R (2017). Clinical immunophenotype of nasopharyngeal neuroendocrine carcinoma with metastatic liver cancer. Clin Chim Acta.

[17] Rizzardi AE, Johnson AT, Vogel RI, Pambuccian SE, Henriksen J, Skubitz AP, Metzger GJ, Schmechel SC (2012). Quantitative comparison of immunohistochemical staining measured by digital image analysis versus pathologist visual scoring. Diagn Pathol.

[18] Lin DC, Meng X, Hazawa M, Nagata Y, Varela AM, Xu L, Sato Y, Liu LZ, Ding LW, Sharma A, Goh BC, Lee SC, Petersson BF, Yu FG, Macary P, Oo MZ, Ha CS, Yang H, Ogawa S, Loh KS, Koeffler HP (2014). The genomic landscape of nasopharyngeal carcinoma. Nat Genet.

[19] You R, Liu YP, Lin DC, Li Q, Yu T, Zou X, Lin M, Zhang XL, He GP, Yang Q, Zhang YN, Xie YL, Jiang R, Wu CY, Zhang C, Cui C, Wang JQ, Wang Y, Zhuang AH, Guo GF, Hua YJ, Sun R, Yun JP, Zuo ZX, Liu ZX, Zhu XF, Kang TB, Qian CN, Mai HQ, Sun Y, Zeng MS, Feng L, Zeng YX, Chen MY (2019). Clonal mutations activate the NF-κB pathway to promote recurrence of nasopharyngeal carcinoma. Cancer Res.

[20] Tay GC, Iyer NG, Ong WS, Tai D, Ang MK, Ha TC, Soo KC, Tan HK (2016). Outcomes and prognostic factors of radiation-induced and de novo head and neck squamous cell carcinomas. Otolaryngol Head Neck Surg.

[21] Tay G, Tan HK, Thiagarajan A, Soo KC, Iyer NG (2014). Squamous cell carcinoma of the ear arising in patients after radiotherapy for nasopharyngeal carcinoma. Eur Arch Oto-rhino-laryngol.

[22] Chan JY, To VS, Wong ST, Wei WI (2014). Radiation-induced squamous cell carcinoma of the nasopharynx after radiotherapy for nasopharyngeal carcinoma. Head Neck.

[23] Giannini L, Incandela F, Fiore M, Gronchi A, Stacchiotti S, Sangalli C, Piazza C (2018). Radiation-induced sarcoma of the head and neck: a review of the literature. Front Oncol.

[24] Kimura H (2018). EBV in T-/NK-cell tumorigenesis. Adv Exp Med Biol.

[25] Tsao SW, Tsang CM, Lo KW. Epstein-Barr virus infection and nasopharyngeal carcinoma. Philos Trans R Soc Lond Ser B Biol Sci 2017; 372(1732). 10.1098/rstb.2016.0270 PMC5597737 28893937

[26] Sturgis CD, Burkey BB, Momin S, Hoschar AP (2015). High grade (large cell) neuroendocrine carcinoma of the nasopharynx: novel case report with touch preparation cytology and positive EBV encoded early RNA. Case Rep Pathol.

[27] Wasserman JK, Papp S, Hope AJ, Perez-Ordóñez B (2018). Epstein-Barr virus-positive large cell neuroendocrine carcinoma of the nasopharynx: report of a case with complete clinical and radiological response after combined chemoradiotherapy. Head Neck Pathol.

[28] Takada K (2012). Role of EBER and BARF1 in nasopharyngeal carcinoma (NPC) tumorigenesis. Sem Cancer Biol.

[29] Rekhtman N, Pietanza MC, Hellmann MD, Naidoo J, Arora A, Won H, Halpenny DF, Wang H, Tian SK, Litvak AM, Paik PK, Drilon AE, Socci N, Poirier JT, Shen R, Berger MF, Moreira AL, Travis WD, Rudin CM, Ladanyi M (2016). Next-generation sequencing of pulmonary large cell neuroendocrine carcinoma reveals small cell carcinoma-like and non-small cell carcinoma-like subsets. Clin Cancer Res.

[30] Miyoshi T, Umemura S, Matsumura Y, Mimaki S, Tada S, Makinoshima H, Ishii G, Udagawa H, Matsumoto S, Yoh K, Niho S, Ohmatsu H, Aokage K, Hishida T, Yoshida J, Nagai K, Goto K, Tsuboi M, Tsuchihara K (2017). Genomic profiling of large-cell neuroendocrine carcinoma of the lung. Clin Cancer Res.

[31] Meder L, König K, Ozretić L, Schultheis AM, Ueckeroth F, Ade CP, Albus K, Boehm D, Rommerscheidt-Fuss U, Florin A, Buhl T, Hartmann W, Wolf J, Merkelbach-Bruse S, Eilers M, Perner S, Heukamp LC, Buettner R (2016). NOTCH, ASCL1, p53 and RB alterations define an alternative pathway driving neuroendocrine and small cell lung carcinomas. Int J Cancer.

[32] Leonetti A, Facchinetti F, Minari R, Cortellini A, Rolfo CD, Giovannetti E, Tiseo M (2019). Notch pathway in small-cell lung cancer: from preclinical evidence to therapeutic challenges. Cell Oncol.

[33] Lim JS, Ibaseta A, Fischer MM, Cancilla B, O’Young G, Cristea S, Luca VC, Yang D, Jahchan NS, Hamard C, Antoine M, Wislez M, Kong C, Cain J, Liu YW, Kapoun AM, Garcia KC, Hoey T, Murriel CL, Sage J (2017). Intratumoural heterogeneity generated by Notch signalling promotes small-cell lung cancer. Nature.

[34] Zhang K, Hong X, Song Z, Xu Y, Li C, Wang G, Zhang Y, Zhao X, Zhao Z, Zhao J, Huang M, Huang D, Qi C, Gao C, Cai S, Gu F, Hu Y, Xu C, Wang W, Lou Z, Zhang Y, Liu L (2020). Identification of deleterious NOTCH mutation as novel predictor to efficacious immunotherapy in NSCLC. Clin Cancer Res.

[35] Alam H, Tang M, Maitituoheti M, Dhar SS, Kumar M, Han CY, Ambati CR, Amin SB, Gu B, Chen TY, Lin YH, Chen J, Muller FL, Putluri N, Flores ER, DeMayo FJ, Baseler L, Rai K, Lee MG (2020). KMT2D deficiency impairs super-enhancers to confer a glycolytic vulnerability in lung cancer. Cancer Cell.

[36] Miao C, et al. Investigating the potential relationship between KMT2C/KMT2D mutations and immune checkpoint inhibitor (ICI) in colorectal cancer (CRC). ESMO Virtual Congress 2020, Abstract 497P.

